# 

**Published:** 1911-06-18

**Authors:** 


### HOSPITAL SUNDAY NUMBER.

### THE HOSPITAL

NEW SERIES. No. 227. Vol. IX.
No. 1,299. Vol. L.

[REGISTERED AS A NEWSPAPER.]

Sunday, June 18, 1911.

